# Utilization of decentralized health facilities and factors influencing women’s choice of a delivery site in Gida Ayana *Woreda*, western Ethiopia

**DOI:** 10.1371/journal.pone.0216714

**Published:** 2019-05-17

**Authors:** Habtamu Tolera, Tegegne Gebre-Egziabher, Helmut Kloos

**Affiliations:** 1 Department of Geography and Environmental Studies, Wollega University, Nekemete, Ethiopia; 2 Department of Geography and Environmental Studies, Addis Ababa University, Addis Ababa, Ethiopia; 3 Department of Epidemiology and Biostatistics, University of California, San Francisco, California, United States of America; University of Botswana, BOTSWANA

## Abstract

**Introduction:**

Despite the government’s efforts to decentralize and expand health institutions to promote facility-based child delivery, home delivery and maternal mortality are still widespread problems in Ethiopia. Most mothers continue to give birth at home. This study aims at identifying the socio-cultural practices, perceived benefit or need, and accessibility factors influencing women’s choice of health facilities for delivery services in Gida Ayana *Woreda*, western Ethiopia.

**Methods:**

We conducted a cross-sectional survey to assess women’s use of delivery care services in Gida Ayana *Woreda*, from November 2016 to January 2017; 459 women who were selected randomly participated in the study. We evaluated the socio-cultural, perceived benefit or need, and economic and physical accessibility factors in women’s choice of delivery care and used adjusted logistic regression analysis to examine significant predictors of delivery site use decisions.

**Results:**

Over half (56.6%) of the women self-reported using institutional delivery care; 80.9% gave birth at a health center. A socio-cultural variable, maternal education, significantly influenced women’s choice of health facility for delivery care services (AOR 3.4; 95% CI 2.0–5.9). Mothers’ knowledge level of obstetric complications and experience of complications during the last birth were the two perceived benefits or need factors associated with higher odds of receiving delivery care from decentralized local facilities. Utilization of health centers for maternal delivery care was significantly higher than of health posts **(**AOR 5.0; 95% CI 2.4–10.2). Availability of motorized transportation during labor to nearby delivery site was a significant predictor of institutional delivery.

**Conclusion:**

This study demonstrates the under-utilization of decentralized health facilities for maternal delivery care services in Gida Ayana *Woreda*, which was significantly influenced by socio-cultural, perceived need, and accessibility factors of women during childbirth. This suggests the need for tailored intervention to improve childbirth services use for mothers in this kind of rural settings.

## Introduction

Childbirth complications remain international public health problems and many thousands of women still die each year from preventable causes before, during, and after giving birth [1–6]. In 2015, such complications resulted in about 275, 000 deaths worldwide, with 99.2% of these occurring in developing countries [4–6]. Sub-Saharan Africa shared the highest burden, accounting for almost half (48.3%) of all maternal deaths globally. Maternal mortality in Ethiopia remains high with a ratio of 497 deaths per 100,000 live births [4,7,8]. The 2015 MMR estimate of the country was 410 per 100,000 live births compared to 373, 338, and 280 in Rwanda, Kenya, and Uganda, respectively [5]. The country reported the largest maternal mortality ratio from all causes for total world deaths [7,8] and did not achieve the goal of driving down maternal deaths to 267 per 100,000 by 2015 [9]. The high maternal death rate was primarily attributed to underutilization of antenatal clinics [10–15] and institutional delivery [16–22]. Several studies indicated that proper follow-up of maternal care can prevent more than 80% of deaths [7,23–26] and others suggest that increasing access to emergency care around the time of delivery is the most essential intervention for driving down maternal deaths [27–34]. According to Woldegiorgis et al [7], only 11.8% of all births were attended by skilled health personnel in Ethiopia, compared to 71.9% in Rwanda and 60.0% in Uganda between 1999 and 2012. Recent surveys noted that skilled birth attendance increased to 28%, far below the national target of 62% by 2015 and 80% by 2020 [9,35,36]. Only 26.2% and 18.8% of women in Ethiopia and Oromia region give birth to their newborns in health facilities respectively [36]. Improving women’s health through institutional delivery has become a major health development agenda of the Ethiopian government since the early 1990s. This is clearly indicated in the new health policy of 1993 and the constitution of the 1995, which argue for the provision of basic maternal healthcare services for women through equitable access to services [37] and decentralization of maternal facilities to nine regions [38]. In 1998, the government provided the free health care initiative for delivery services. In 2002, local decentralization fully empowered *woredas* (districts) governments to manage maternal care through a community-level primary healthcare system: *woreda* (district) hospital, health center and health post [35]. Each peripheral health post is staffed with two female health workers who assist village women to use delivery center [39,40].

Despite measures to increase the number of women delivering at health facility, underutilization of local primary health facilities reduces the effectiveness of the measures. A knowledge of the factors associated with low institutional delivery is critical for identifying gaps in the existing research, planning interventions, and developing effective policies for addressing the problem [41,42]. Studies have been conducted to address the socio-demographic and facility level determinants of delivery care [25,43,44]. Several studies also indicated that these components may not be enough for a mother to seek or to reach delivery care [25,28,45,46]. It is known that utilization of maternal services is also a function of sets of cultural practices, perceived benefit or need, economic and physical accessibility [24,45,46]. There is however a paucity of evidence on the specific socio-cultural, perceived benefit or need, healthcare availability and accessibility factors to explain the utilization of delivery care in Ethiopia [25,28,45,47]. Moreover, the available studies on factors that influence women’s use of health facility are based on secondary data from the health facility [28,47]. This study uses primary data to analyze the utilization of decentralized health facilities and the socio-cultural, perceived benefit or need, healthcare availability and accessibility factors associated with women’s choice of delivery site [16–19,42,48].

## Methods

### Study design and period

A household-based cross-sectional study was carried out in Gida Ayana *Woreda* (District) to examine utilization of decentralized health facilities for childbirth and factors associated with women’s choice of delivery sites.

### Study setting

The study was carried out in four *kebeles* (the smallest administrative unit in Ethiopia) of Gida Ayana *Woreda*, Eastern Wollega Zone, Oromia Region, in western Ethiopia. The *woreda* had a total population of about 140,000 in 2017; 78.1% were rural residents [49–51]. The four *kebeles* studied were Ayana, Ejere, Angar, and Lalistu. The *woreda* is primarily inhabited by the Oromo ethnic group with small numbers of Amhara and Tigre [50,51]. The *woreda* has 1 primary hospital, 5 health centers, and 28 health posts. Roads are scarce in the *woreda* and there is no road in the study *kebeles*. People walk, use donkeys, or horses, and motorcycles to travel. As a result, it takes several hours of walking to reach the nearest paved road and motorized transport to public health delivery sites. Stretchers carried by men are used to transport women to the nearest motorway [51].

### Study variables

#### Outcome variable

The outcome variable was health facility use for childbirth service among reproductive women aged 15 to 49 years who gave last birth during the 5 years prior to this study. The variable was coded 1 if the women chose a health institution for delivery of their most recent birth; otherwise, delivery at home was coded 0.

#### Predictor variables

The explanatory variables were socio-cultural, benefit or need, and accessibility characteristics of women. According to expanded ‘‘three delays model” [24,46], socio-cultural, perceived benefit or need, economic and physical accessibility characteristics affect women’s choices of maternal facility for delivery use. These are the main causes of phase I and II delays while phase III delay are factors that affect women in receiving adequate care at the health facility. The phase III delay factors are not considered in this study. Studies further classified the factors as individual and health facility-related factors. The individual factors include the mothers’ socio-cultural factors and the perceived benefit or need of facility use. Both factors directly influence mothers’ decision to seek care (Phase I delay). Economic and physical accessibility factors are phase II delays that determine whether the woman actually identifies and reaches the health facility [18]. Perceived accessibility also influences decision-making for delivery (Phase II delays) [18].

The socio-cultural factors that affect pregnant women’s decision to seek delivery services include maternal age, marital status, ethnicity, religion, traditional beliefs, family composition, women’s autonomy, women’s and husband’s education [28,46,47]. Perceived benefits or needs include maternal information availability, maternal health knowledge, desire of pregnancy, perceived quality of care, antenatal care (ANC) use, previous delivery service use, birth order, and pregnancy complications and other factors related to women’s perceptions of the benefit of facility delivery and the need to seek delivery care [46]. Several studies identified economic accessibility factors as financial capability and opportunity costs which include mother’s occupation, husband’s occupation and the ability to pay and physical accessibility factors as region, place of residence, distance, availability of transportation services and health facility [28,46].

The socio-cultural practice and perceived benefits or needs considered in our study were maternal age, education, autonomy of decision to deliver at health facilities, support of traditional practice of the society for women to deliver at facilities, whether delivery at a health facility is considered to be necessary, plans to deliver at facilities, number of births, ANC clinic visits, knowledge of obstetric complications, presence of complications during earlier and last births, possession of a radio and/or TV at home, knowledge of free services, home visits by community health agents, and women meetings attendance. Maternal occupation and monthly household income were included as economic factors. Maternal residence, distance to health facilities, availability of motorized transport, and types of decentralized local health facilities for birth were the physical accessibility factors included in the study.

### Inclusion and exclusion criteria

Women aged 15–49 years living in the study *kebeles* for at least 5 years prior to the survey were included in the study and women with mental illness were excluded.

### Operational definitions

#### Home delivery

Giving birth in the mother’s home or the home of a neighbor, relative, or member of her extended family.

#### Institutional delivery

Giving birth at a health facility, including a health post, health center, hospital, or private clinic.

#### Woman’s autonomy

Having the ability to decide either by herself or with her husband where to deliver her last child.

#### Decentralized facilities

Health institutions, including health posts, health centers, primary hospitals and private clinics that were established to serve the community in Gida Ayana *Woreda* since the implementation of the national *woreda* decentralization program in 2002.

#### Home visit

Household-based visit made by health extension workers during last birth.

#### Women meetings

Monthly meetings of pregnant women at the health post level.

### Study population and sampling

The source population for this study was all women of reproductive age (15 to 49 years) living in Gida Ayana *Woreda*, western Ethiopia and the study subjects were women aged 15 to 49 years who had their last birth during the 5 years preceding the survey. For sample size estimation, we assumed a 95% confidence interval (CI), a margin of error of 5%, a proportion of 33% utilizing public health services [52], and a design effect of 1.5. We used a design effect because we employed the two-stage sampling method. In the first stage of sampling, the four *kebeles*: Ayana, Angar, Ejere, and Lalistu were randomly selected using the lottery system. At the second stage of sampling, study participants were selected by simple random-number sampling from the respective *kebeles* proportional to their population size. With these considerations, the minimum adequate sample size was computed based on the statistical estimation method [53]. Since the source population was estimated to be less than 10,000, the sample size was corrected. A 5% non-response rate was used to obtain the final sample size of 459. All the selected women gave written consent to participate in the study. Five women wanted to end the interviews early due to personal appointments they had to attend to; they were reported as non-responders.

### Data collection and quality control

Data were collected in paper from mothers at households between November 2016 and January 2017 through an interviewer-administered structured questionnaire. A structured questionnaire was adapted from the Demographic Health Survey and other similar works of literature. The questionnaire was initially designed in English, based on the purpose of the study, translated to the local language, Afan Oromo, for the interviews, then translated back to English by experts for consistency. The questionnaire contained socio-cultural, perceived benefit/need, and economic and physical accessibility questions pertaining to maternal service use.

Eight local female interviewers were recruited for data collection. All of them were experienced in data collection, hold bachelor of science degrees or diplomas in health science and could speak the local language. Two nurses experienced in supportive supervision were recruited as supervisors. All interviewers and supervisors were trained for 2 days by the first author on the objectives, instruments, and ethics of the study. A pilot survey of 10% of the study population was carried out to test the questionnaire. The supervisors and the principal investigator supervised and monitored data collection activities and checked all the completed questionnaires for consistency and missing data daily. Incomplete questionnaires deemed to have problems were returned to the interviewers for completion. A final check was made during data entry through double data entry using EpiData version 3.1 (EpiData; CDC, 2000). The accuracy of data entries was verified using two methods. First, 10% of the questionnaires were randomly selected and checked. Second, frequency distributions, descriptive statistics, and results from cross-tabulations were carefully checked before fitting logistic regressions.

### Data processing and analysis

The SPSS Version 24.0 was used for data processing and analysis. The analysis employed logistic regression to study the effect of the independent variables on the dependent variable by controlling confounders. The Hosmer-Lemeshow test was used to compare and examine the goodness of fit of the model [54]. Multicollinearity was also examined, and all covariates having a value of variance inflation factor of less than 5% were retained in the analysis. The strength of association was assessed using adjusted odds ratios with a 95% confidence interval (CI) at a *p*-value less than 0.05.

### Ethical considerations

The study was approved by the Research Ethics Review Committee (RERC) of Wollega University with a reference letter numbered of WU99529-H1-3-24/11/2016. Permission was received from Gida Ayana *Woreda* Health Office. The purpose of the study was explained to all participants and a consent form approved by the Review Board was given to participants. Parents or legal guardians provided consent for all participants under age 18. We emphasized that participation was completely voluntary and that they had the right to withdraw any time during the interview without giving any reason. Confidentiality and anonymity were explained to all participants. We ensured that all participants understood the information given by asking them. The consent form was read aloud for women who could not read or write. Literate women were asked to read the consent form. A written consent in the form of a signature or a thumbprint was obtained from all of the participants. None of the participants refused to be interviewed. Five women wanted to end the interviews early due to personal appointments they had to attend to; they were reported as non-responders.

## Results

### Socio-cultural and demographic backgrounds of women

The mean and standard deviation of mothers’ age was 26.1 and 7.1 years, respectively; 56% were in the age group of 20 to 34 years. Almost half of the respondents reported that they were illiterate, 48.9% were Oromo ethnic group, 57.3% were Christians and the majority of participants were married. Over half of the participants reported that their society supported institutional delivery traditionally, and 88.1% of women considered institutional delivery to be necessary (Table 1).

**Table 1:** Characteristics of study participants, Gida Ayana, western Ethiopia.

Variable	*n(%)*
**Socio-cultural and demographic characteristics**	
Maternal mean age (years)	26.1 ± 7.1 SD
Maternal marital status	
Married	381(83.9)
^ᵃ^Other	73(16.1)
Maternal ethnicity	
Oromo	222(48.9)
^b^Other	232(51.1)
Maternal religion	
Christian	260(57.3)
Moslem	194(42.7)
Maternal literacy	
Can read and write	230(50.7)
Illiterate	224(49.3)
Support of traditional practice for facility care	
Yes	246(54.2)
No	208(45.8)
Delivery at a health facility is necessary	
Yes	400(88.1)
No	54(11.9)
**Perceived obstetric need and knowledge of women**	
ANC visit for the last pregnancy	
Yes	294(64.8)
No	160(35.2)
Plan to deliver at a facility	
Yes	190(41.9)
No	264(58.1)
Knowledge of pregnancy, labor and birth complications	
Yes	241(53.1)
No	213(46.9)
Complications during the previous birth	
Yes at least one	256(56.4)
No any	198(43.6)
Use of health facilities for the last birth	
Yes	257(56.6)
No	197(43.4)
Assistance during delivery at home (*N* = 197)	
TBA	101(51.3)
Relative or neighborhood	92(46.7)
Skilled person	4(2.0)
Attended monthly women meetings	
Yes	142(31.3)
No	312(68.7)
A home visit by the health extension worker	
Yes	199(43.8)
No	255(56.2)
Knowledge of free service for delivery care	
Yes	238(52.4)
No	216(47.6)
Mean number of births	3.1 ± 2SD
Possession of radio and/or TV	
Yes	243(53.5)
No	211(46.5)
**Economic and physical accessibility**	
Maternal occupation	
^c^Non-housewife	256(56.4)
Housewife	198(43.6)
Mean monthly household income ($US)	47 ± 15.1SD
Mean walking distance to the delivery site (minutes)	51.1 ± 32SD
Maternal residence	
Urban	254(55.9)
Rural	200(41.1)
Availability of motorized transport	
Easily available	145(31.94)
Difficult	309(68.06)
**Utilization of decentralized health facilities**	
Delivery by type of health facility (*N* = *257*)	
Hospital or clinic	22(8.6)
Health centers	208(80.9)
Health posts	27(10.5)
Delivery in a health facility by *kebele* (*N* = 257)	
Ayana	77(81.9)
Ejere	46(59.7)
Lalistu	56(45.5)
Angar	78(48.8)

^ᵃ^Other refer to single, divorced or widowed women.

^b^Others include Amhara and Tigre.

^c^Non-housewife activities include skilled employment, small business/service, and farming.

ANC: Antenatal care. SD: Standard Deviation. $US: United States Dollars with the exchange value of 27 Ethiopian Birr (Nov. 2016). TV: Television. TBA: Traditional birth attendant.

### Perceived need characteristics and knowledge of women

The mean number of children born to a mother was 3.1 (ranging from 1 to 6) with a standard deviation of 2. Almost two-thirds of the participants used ANC services and 41.9% had plans to deliver at local health facilities in the future. More than half of the mothers had reportedly been informed about potential pregnancy, labor, and delivery obstetric complications during the last birth; 56.4% had encountered at least one complication during previous births, and 56.6% reported they had faced at least one delivery complication during the last birth. Thirty-one percent of women attended monthly meetings for pregnant women, 43.8% had been visited by a community health agent, and 52.4% were well informed about the availability of free delivery services through the free care initiative for pregnant women. Of the 197 mothers delivering at home, 51.3% reported they were assisted by a traditional birth attendant.

### Economic and physical accessibility characteristics of women

Forty-four percent of participants were housewives. The mean monthly income of the mothers’ households was 47.0 $US (around 1, 270 Ethiopian Birr) with a standard deviation of 15.1 $US. The mean and standard deviation for walking to the health facilities for delivery care were 51.1 and 32.0 minutes, respectively.

### Utilization of decentralized health facilities for delivery care

Of the 459 mothers who participated in this study, 98.9% provided valid answers to the questionnaire. Over half of the mothers reportedly had their last child delivered in a health facility; 43.4% delivered at home. In regard to women’s choice of child delivery site across decentralized health systems, 80.9% reported that they gave birth at local health centers, 10.5% at health posts, and 8.6% in the local hospital or in a private clinic. The majority of women in Ayana and Ejere *kebeles* preferred health facilities, and most participants in Lalistu and Angar reportedly gave birth at home.

### Factors influencing women’s choice of the delivery site

A model adjusted for confounding factors maternal age at delivery, number of births, maternal literacy, support of traditional practice, ANC clinic visit, plan to deliver at a facility, maternal knowledge of pregnancy, labor, and birth complications, complications during last birth, home visit by community health agents, availability of motorized transport during labor and type of health facility for delivery care (Table 2). The confounding factors were determined using before and after adjustments. Adjusted Odds Ratio (AOR) of socio-cultural, perceived benefit/need, and accessibility factors predicting women’s choice of the delivery site were presented as follows:

#### Socio-cultural factors

After adjustment for confounding factors (Table 2), the results of the multivariable logistic regression analysis showed that literate mothers were 3.4 times more likely to deliver in health facilities (AOR 3.4; 95% CI 2.0–5.9) than illiterate mothers. Women who reported that the traditional practices of the society were positive towards institutional delivery were almost 3 times more likely to give birth at health facilities compared to their counterparts who reported that the tradition of their society did not support delivery at health facilities (AOR 2.9; 95% CI 1.7–4.9).

#### Perceived benefit or need factors

In a multivariate model, mothers who had plans to deliver in nearby health facilities in case of complications had almost 2 times higher odds of giving birth at health facilities (AOR 1.8; 95% CI 1.1–3.0) than those who had no plans. Participants who knew about obstetric complications related to pregnancy, labor, and delivery were 2.5 times more likely to deliver at facilities (AOR 2.5; 95% CI 1.4–4.2) compared to the reference group, and those who experienced at least 1 complication during the last birth were almost 3 times more likely to give birth at health facilities (AOR 2.9; 95% CI 1.6–5.2) than women who reported no complications. Mothers who were visited by community health agents during pregnancy period were nearly twice as likely to deliver at health facilities than those who were not visited (AOR 1.7; 95% CI 1.1–2.9).

#### Accessibility factors

In a multivariate analysis, women who used health centers were 5 times more likely (AOR 5.0; 95% CI 2.4–10.2) than those who used health posts. Our study also found that mothers who reported ease of access to motorized transport in their last birth were nearly twice as likely to deliver their child at a healthcare facility than their counterparts who faced difficulties in securing motorized transportation to the delivery site (AOR 1.9; 95% CI 1.1–3.3).

**Table 2:** Factors influencing women’s choice of delivery sites, Gida Ayana *Woreda*, western Ethiopia.

	Delivery site choice			
Variable	Health facility (*N =* 257)	Home (*N =* 197)	COR(9%CI)	AOR(9%CI)
**Socio-cultural factors**				
Maternal age (in years)				
19 or less	82(64.6)	45(35.4)	2.2(1.2˗4.1)*	0.9(0.4˗2)
20˗34	143(56.1)	112(43.9)	1.5(0.9˗2.7)	0.9(0.4˗1.8)
35 or more	32(44.4)	40(56.6)	1	1
Number of births				
0˗1	139(64.4)	77(35.6)	2.1(1.2˗3.4)*	0.9(0.4˗2.0)
2˗3	77(51.7)	72(48.3v	1.2(0.7˗2.1)	0.6(0.3˗1.4)
4 or more	41(46.1)	48(53.9)	1	1
Maternal literacy				
Can read and write	167(72.6)	63(27.4)	3.9(2.6˗5.8)**	3.4(2.0˗5.9)**
Illiterate	90(40.2)	134(59.8)	1	1
Autonomy to discuss with husband				
Yes	193(59.0v	134(41.0)	1.4(0.9˗2.1)	
No	64(50.4v	63(49.6)	1	
Support of traditional practice				
Yes	167(67.9v	79(32.1)	2.7(1.8˗4.0)**	2.9 (1.7˗4.9)**
No	90(43.3v	118(56.7)	1	1
Delivery at a health facility is necessary				
Yes	231(57.8)	169(42.2)	1.4(0.8˗2.6)	
No	26(48.1)	28(51.9)	1	
**Perceived need factors**				
ANC clinic visit				
Yes	198(67.3)	96(32.7)	3.5(2.3˗5.2)**	1.5(0.8˗2.7)
No	59(36.9)	101(63.1)	1	1
Plan to deliver at a facility				
Yes	126(66.3)	64(33.7)	1.9(1.3˗2.9)**	1.8(1.1˗3.0)*
No	131(49.6)	133(50.4)	1	1
Knowledge of pregnancy, labor and birth complications				
Yes	173(71.8)	68(28.2)	3.9(2.6˗5.7)**	2.5(1.4˗4.2)*
No	84(39.4)	129(60.6)	1	1
Complications during previous births.				
At least one	145(56.6)	111(43.4)	1.0(0.6˗1.4)	
None	112(56.6)	86(43.4)	1	
Complications during the last birth				
At least one	221(63.5)	127(36.5)	3.3(2.1˗5.3)**	2.9(1.6˗5.2)**
None	36(34.0)	70(66.0)	1	1
Attended monthly women’s meetings				
Yes	97(68.3)	45(31.7)	2.0(1.3˗3.1)*	1.6(0.9˗2.7)
No	160(51.3)	152(48.7)	1	1
A home visit by community health workers				
Yes	139(69.8)	60(30.2)	2.6(1.8˗3.9)**	1.7(1.1˗2.9)*
No	118(46.3)	137(53.7)	1	1
Knowledge of free service for childbirth				
Yes	141(59.2)	97(40.8)	1.2(0.8˗1.8)	
No	116(53.7)	100(46.3)	1	
Possession of radio and/or TV				
Yes	155(63.8)	88(36.2)	1.8(1.2˗2.7)*	1.0(0.6˗1.7)
No	102(48.3)	109(51.7)	1	1
**Economic accessibility factors**				
Average monthly household income				
50 $US or more	153(56.0)	120(44.0)	0.94(0.6˗1.3)	
Less than 50 $US	104(57.5)	77(42.5)	1	
Maternal occupation				
Non-housewife	164(64.1)	92(35.9)	2.0(1.3˗2.9)**	1.5(0.9˗2.5)
Housewife	93(47.0)	105(53.0)	1	1
**Physical accessibility factors**				
Maternal residence				
Urban	155(61.0)	99(39.0)	1.5(1.0˗2.1)*	0.4(0.2˗1.8)
Rural	102(51.0)	98(49.0)	1	1
Walking distance to delivery site in minutes				
Less than 30	174(58.8)	122(41.2)	1.2(0.8˗1.9)	
30 or more	83(52.5)	75(47.5)	1	
Availability of motorized transport				
Easily available	100(69.0)	45(31.0)	2.1(1.4˗3.2)**	1.9(1.1˗3.3)*
Difficult	157(50.8)	152(49.2)	1	1
Type of delivery or decentralized health facility				
Hospital or clinic	22(61.1)	14(38.9)	7.1(3.2˗15.7)**	
Health center	208(77.6)	60(22.4)	15.7(9.5˗26)**	
Health post	27(18.0)	123(82.0)	1	

1: reference category.

**significant at *p* < 0.01.

* significant at *p* < 0.05. CI: Confidence Interval. COR: Crude Odds Ratio. AOR: Adjusted Odds Ratio.

## Discussion

Although the level of utilization of decentralized maternal childbirth services in Gida Ayana *Woreda* was 56.6%, more than twice as high as the national figure (26.2%), it is still low compared to other developing countries. Results showed that socio-cultural characteristics of maternal literacy and the support of traditional community practice for delivery use; perceived benefit or need factors of plan to deliver at a facility, knowledge of complications during pregnancy, labor and birth, complications during last birth, and home visits by community health agents were significantly associated with women’s utilization of maternal health facilities for delivery service. Multivariate analysis indicates that women with physical access to motorized local transportation services during labor and access to the preferred type of health facility were more likely to seek care from health facilities to deliver their last child. However, the study results are inconclusive with respect to the influence of economic accessibility, maternal occupation and household monthly income on the use of maternal delivery care services.

Women who could read and write were more likely to receive institutional delivery care services from skilled providers than those with no education. Women’s choice of institutional delivery is strongly influenced by education. This finding is corroborated by other studies [1,27,45,55–57]. The possible explanation for this finding is that educated women may be more aware of pregnancy complications and delivery at home and may know the importance of delivering with the help of a skilled provider.

This study also found that the support of traditional birthing practices of the local community positively influenced the odds of women delivering at health facilities compared to women who reported that the traditions of their communities did not allow them to use institutional delivery services. Cultural beliefs and practices influence their choices of health care providers in the study setting, corroborating the findings of other studies [11,24,28,41,42,46].

In developing countries, the use of public health delivery services has been found to be significantly higher among women who had information about the risk factors of pregnancy, labor, and delivery during their last birth [22,24,26,42–46,58–61]. These findings are similar to our results, which show that women who had information about obstetric complications during the last birth were 2.5 times more likely to deliver at health facilities than their counterparts who did not have that information. The strong relationship between education and delivery in public health facilities appears to be linked to knowledge about the risks of delivering at home and the greater safety of skilled delivery care.

Our findings also show that maternal plans to give birth at health facilities exerted a significant influence on women’s choice of delivery care services, after controlling for selected covariates. Women who had planned to give birth at health facilities in case of complication were almost 2 times more likely to deliver at a birth center than women who did not have a birth plan. Studies in Asia and Africa have documented that women who saved money and made arrangement for transportation and potential birthing risks were more likely to use public delivery services [1,16,22,23], pregnant women without delivery plans either used unsafe childbirth methods or gave birth at home [41,42,54,58,62].

Institutional delivery was nearly 3 times more prevalent among women who experienced at least one complication during their last pregnancy, mostly during labor, than among women who did not report any complication. This indicated that mothers were not likely to use skilled delivery services unless they had experienced complications. This could be due to the poor knowledge of potential complications and lack of a birth preparedness plan, cultural influence, or poor counseling during pregnancy [7,24,43,45,46]. Also in the current study, of the total women who gave the most recent birth to a child in the Gida Ayana *Woreda*, it was only 41.9% who prepared a plan to give birth at a health institution. The majority of women did not plan to use health facility during delivery. This scenario may be incorporated into health promotion messages aimed at overcoming traditional attitudes of complacency and other forms of aversion to the use of professional delivery services.

The type of health facility significantly predicted the odds of delivery at a facility. Our multivariate analysis showed that the decentralized healthcare system enhanced the uptake of local delivery services. The odds of choosing decentralized health center for delivery than a health post were 5 times higher when other variables were controlled. This finding corroborates studies done in Guji, Wolaita, and Dawaro zones, Dembecha *Woreda*, and in a countrywide survey in Ethiopia [7,43,55,60], Kenya, Jordan [63,64]. However, unlike these studies, in rural Rwanda over 90% of births were recorded in health posts where decentralized delivery care extended effectively to the health posts [40]. Decentralization facilitated intensive community health worker education and mobilization, provision of clean newborn clothes at the time of delivery, and financial disincentives for home delivery, thus significantly increasing positive delivery outcomes [26,40].

The positive relationship between home-based counseling by local health providers and utilization of healthcare services is well documented [11,13,42,43,57,61,63]. Similarly, home visits by community health agents during last pregnancy were strongly associated with the outcome variable in Gida Ayana. Women who were visited opted for facility delivery care services whereas most women who were not visited preferred to give birth at home. The explanation might be that well-informed women in Gida Ayana are more aware of health risks and obtain delivery at health facilities.

Availability of motorized transport was found to be another major factor in the choice of delivery. Studies carried out elsewhere found higher odds of health facility use by women who have access to better local transport systems or road connections [43,45,46,56–58,63,65]. Our multivariate analysis indicated that women having some form of motorized local transport services have higher odds of accessing and using maternal healthcare facilities during the last labor compared to those with difficult access to transport service.

## Strengths and limitations

As a strength, this study utilized a relatively large and randomized sample, increasing the power of the study and making the results broadly representative. However, this study has some limitations. Primarily, women might have had difficulties of remembering maternal information from the 5 years preceding our study, causing recall bias. Secondly, of the ‟three delays model”, the third delay namely receiving adequate care in the facility is not addressed in the current study. Data on the quality of delivery services and information related to waiting time at the delivery sites were not collected. Health facility-related factors may have prevented some women from utilizing the health services for delivery. We recommend a study of factors influencing the utilization of decentralized health facility after stratified analysis with interaction terms and sampling weights employing small risk factors of delivery care service use in western Ethiopia.

## Conclusion and recommendations

Many underlying socio-cultural, perceived benefit or need, and physical accessibility factors were the significant predictors of women’s use of health facility. This study found that maternal education influenced women’s attitudes toward the use of health facilities for delivery services. Therefore, policymakers need to implement outreach interventions through *kebeles* to encourage women to use services. It is also crucial for health promotion programs to target rural women and communities with different traditional practices and views about the importance of knowledge on potential pregnancy, labor and delivery complications. The ministry of health, local government authorities and health care services, childbirth professionals and community health extension agents can contribute in this regard. Equally important, is intervention program to raise awareness and community mobilization campaigns to overcoming the deep seated cultural aversion of using delivery services in public health facilities. In addition, women need to plan to deliver at health facilities to overcome complications and increase their ability to give safe birth. This has to be supported with increasing the accessibility of delivery care clinics either through further decentralization of health centers or by upgrading the frontline health posts into health centers, as well as implementing community-based intervention programs and improving rural transport services and the quality of maternal services.

## Supporting information

S1 Table Sample distribution from the study communities by sample *kebele* (sub-district). Description of data: Sample units drawn for the study among the studied *kebeles*.
(DOCX)

S1 Text English language questionnaire for study entitled “Utilization of decentralized health facilities and factors influencing women’s choice of a delivery site in Gida Ayana *Woreda*, western Ethiopia”. Description of data: The questionnaire used to collect data for the study.
(DOCX)

S1 Data Some parts of data used in this study to measure the utilization of a decentralized health facilities for delivery care in Gida Ayana *woreda*, western Ethiopia. (XLSX)
