# Supplementary material for: Utilization of decentralized health facilities and factors influencing women’s choice of a delivery site in Gida Ayana Woreda, western Ethiopia
Source: PLoS One. 2019 May 17;14(5):e0216714. doi: 10.1371/journal.pone.0216714 (PMC6524803; doi:10.1371/journal.pone.0216714)
Supplement: S1 Table — Description of data: Sample units drawn for the study among the studied kebeles. (DOCX) [file pone.0216714.s001.docx]

**Supplementary Table 1:** Sample distribution from the study communities by sample *Kebeles.*

| ***Kebele* (Sub-district)** | **Total women in reproductive age who gave birth to child five years ago preceding the survey** | **Sample size** |
| --- | --- | --- |
| Ayana | 410 | 94 |
| Ejere | 338 | 77 |
| Angar | 719 | 163 |
| Lalistu | 550 | 125 |
| **Total** | 2017 | 459 |
