# Supplementary material for: Utilization of decentralized health facilities and factors influencing women’s choice of a delivery site in Gida Ayana Woreda, western Ethiopia
Source: PLoS One. 2019 May 17;14(5):e0216714. doi: 10.1371/journal.pone.0216714 (PMC6524803; doi:10.1371/journal.pone.0216714)
Supplement: S1 Text — Description of data: The questionnaire used to collect data for the study. (DOCX) [file pone.0216714.s002.docx]

**Household Survey Questionnaire for women**

**Addis Ababa University**

**College of Social Sciences**

Questionnaire on assessment of utilization of decentralized health facilities and factors influencing women's choice for delivery care site in Gida Ayana *Woreda*, Oromia Region, western Ethiopia.

**Consent form that certify the respondents agreement before the interview**

1. Name of the *Kebele*:__________________________________________
2. Questionnaire Identification Number:________________________

**Introduction**

Good morning, (or Good afternoon). My name is _____________________. I am public health science professional and now I am collecting data from pregnant women of reproductive age groups (15- 49 years) for the research being conducted to identify factors affecting utilization of institutional delivery service utilization and associated factors in Gida Ayana *Woreda*, western Ethiopia: a community based cross sectional study by Mr. Habtamu Tolera, and Professor Tegegne Gebre Egziabher from Addis Ababa University Social Sciences College and Dr. Helmut Kloos from California University, Department of Epidemiology and Biostatistics. You are selected to be one of the participants in the study by chance. The study will be conducted through interview. Your name and other personal identifiers will not be recorded on data collection format and the information that you give us will be kept confidential and will also be used for this study purpose alone. A code number will identify every participant and no names will be used. If a report of the result is published, only summarized information of the total group will appear. The interview takes 30 minutes on average and is voluntary and you have the right to participate, or not to participate or to refuse at any time during the interview. You will not face any problem if you do not agree to the information to be asked. Your participation on this study helps to improve and identify factors affecting institutional delivery service utilization in Gida Ayana *Woreda*, Oromia Region, western Ethiopia. If you have any questions about this study you can ask me or contact the principal investigator Mr. Habtamu Tolera

Email Address: habtol@yahoo.com.

Mobile: +251912015545

P.O.Bo:1176

Are you willing to participate in the study?

1. Yes 2. No

Interviewer who certified that the informed consent has been given in written from the respondents

Name:__________________ Signature:__________________________Date:_______________

**Result**

1. Completed
2. Partially completed
3. Refused to complete

**Checked by:**

Name:______________________________Signature:__________________Date:__________

**Instruction**: For the questions that have alternatives, encircle to the response of the mother. Write appropriate response (s) on the space provided for questions for which alternatives are not given.

**Thank you in advance for your cooperation!**

**Part I: Socio-cultural and demographic haracteristics of women**

| **S/N** | **Questions** | **Response option** | **Skip** |
| --- | --- | --- | --- |
| 101 | Age of mother at last birth | Age in complete years [_________________] |  |
| 102 | What is your marital status? | Single-------------------------------------0  Married ----------------------------------1  Divorced---------------------------------2  Widowed---------------------------------3  Others [specify] ------------------------99 |  |
| 103 | What is your religion? | Protestant---------------------------------0  Orthodox---------------------------------1  Muslim-----------------------------------2  Catholic----------------------------------3  Other [Specify] ------------------------99 |  |
| 104 | What is your ethnicity? | Oromo------------------------------------0  Amhara ----------------------------------1  Tigire-------------------------------------2  Other [Specify]-------------------------99 |  |
| 105 | The number of children a woman gave birth to? | Number[__________________________] |  |
| 106 | Maternal education | Cannot read and write -------------------0  Able to read and write -------------------1  Primary school [1-8] ---------------------2  Secondary school [10-12] ---------------3  College diploma and above -------------4 |  |
| 107 | Did you have the right to discuss with your husband or partner to fix the delivery site for the last birth? | Yes-----------------------------------------------0  No------------------------------------------------1 |  |
| 108 | Support of traditional practice to use local health facility for delivery care during last pregnancy | Yes-----------------------------------------------0  No------------------------------------------------1 |  |
| 109 | Considered delivery at a health facility to be necessary | Yes-----------------------------------------------0  No------------------------------------------------1 |  |

**Part II: Women's perceived obstetric history and knowledge of maternal health care**

| **S/N** | **Questions** | **Response** | **Remark** |
| --- | --- | --- | --- |
| 201 | Where did you give birth to your last child? | Health institution------------------------0  Home-------------------------------------1  If other [specify]-----------------------99 |  |
| 202 | Did you have Antenatal visits to health facility during your last delivery? | Yes----------------------------------------0  No-----------------------------------------1  If other [specify]-----------------------99 |  |
| 203 | Did you have birth plans to deliver at a facility in case of complication during last birth? | Yes----------------------------------------0  No-----------------------------------------1  If other [specify]--------- -------------99 |  |
| 204 | Have you had a knowledge of pregnancy, labor, and birth complication during last birth? | Yes----------------------------------------0  No-----------------------------------------1 |  |
| 205 | Have you had any health problems during previous pregnancy | Yes, at least one ------------------------0  No, without any complication--------1 |  |
| 206 | Have you faced any health problems during last birth? | Yes, at least one ------------------------0  No, without any complication--------1 |  |
| 207 | Do you attend monthly a held pregnant women’s meetings during your last pregnancy? | Yes----------------------------------------0  No-----------------------------------------1 |  |
| 208 | Do the community health workers offer you home counseling during last birth? | Yes----------------------------------------0  No-----------------------------------------1 |  |
| 209 | Do you have the knowledge of free service for childbirth during last pregnancy? | Yes----------------------------------------0  No-----------------------------------------1 |  |
| 210 | Use of health facilities for the last birth | Yes----------------------------------------0  No-----------------------------------------1 |  |
| 211 | Assistance during delivery at home | TBA  Relative or neighbor  Skilled person |  |
| 212 | Do you have radio/TV in your home? | Yes----------------------------------------0  No-----------------------------------------1 |  |

**Part III: Women's economic and physical accessibility characteristics to use health facilities**

| **S/N** | **Question** | **Response** | **Remark** |
| --- | --- | --- | --- |
| 301 | Estimated household monthly income? | in Ethiopian Birr:[_________________] |  |
| 302 | What is maternal occupation during last birth? | Housewife----------------------------------0  Government Employed.-------------------1  Small business/service---------------------2  Farmer---------------------------------------3  If other [specify]-------------- ----------99 |  |
| 303 | Location of maternal residence? | Urban----------------------------------------0  Rural-----------------------------------------1 |  |
| 304 | Maternal *Kebele* | Write name your *kebele* [____________] |  |
| 304 | Estimated walking distance to closest delivery site? | In hours:[______________________] |  |
| 305 | How did you judge the availability of motorized transport service during your last labor? | Easily available ----------------------------0  Difficult--------------------------------------1 |  |
| 306 | Decentralized health facility available nearby your home during last birth? | Hospital or clinic---------------------------0  Health center--------------------------------1  Health post----------------------------------2  If other [specify]-------------- -----------99 |  |
| 307 | Place of delivery for last pregnancy? | Hospital or clinic---------------------------0  Health center--------------------------------1  Health post----------------------------------2  Home----------------------------------------3 |  |

**...................................END......................................................**

**Thank you once again!**
